# Characteristics and Risk Factors Associated with SARS-CoV-2 Pneumonias in Hospitalized Pediatric Patients: A Pilot Study

**DOI:** 10.3390/children10101703

**Published:** 2023-10-19

**Authors:** María Hernández-García, Claudia Solito, Alba Pavón Ortiz, Noelia Arguedas Casamayor, Maria Melé-Casas, Gemma Pons-Tomàs, Mariona F. de Sevilla, Rosa Pino, Cristian Launes, Carmina Guitart, Mònica Girona-Alarcón, Iolanda Jordan, Juan José García-García

**Affiliations:** 1 Paediatrics Department, Hospital Sant Joan de Déu Barcelona, 08950 Barcelona, Spain; maria.hernandezg@sjd.es (M.H.-G.); claudia.solito@sjd.es (C.S.); alba.pavon@sjd.es (A.P.O.); noelia.arguedas@sjd.es (N.A.C.); maria.mele@sjd.es (M.M.-C.); gemma.pons@sjd.es (G.P.-T.); mariona.fernandez@sjd.es (M.F.d.S.); rosamaria.pino@sjd.es (R.P.); cristian.launes@sjd.es (C.L.); juanjose.garciag@sjd.es (J.J.G.-G.); 2 Infectious Diseases and Microbiome, Institut de Recerca Sant Joan de Déu (IRSJD), 08950 Barcelona, Spain; carmina.guitart@sjd.es (C.G.); monica.girona@sjd.es (M.G.-A.); 3 Faculty of Medicine and Health Sciences, Universitat de Barcelona, 08036 Barcelona, Spain; 4 Centro de Investigación Biomédica en Red de Epidemiología y Salud Pública (CIBER-ESP), Instituto de Salud Carlos III, 28029 Madrid, Spain; 5 Paediatric Intensive Care Unit, Hospital Sant Joan de Déu Barcelona, 08950 Barcelona, Spain

**Keywords:** SARS-CoV-2, COVID-19, pneumonia, pediatrics, risk factors

## Abstract

SARS-CoV-2 pneumonia in children has a lower incidence and severity compared to adults. Risk factors are adolescence and comorbidities. Our aims were to describe the characteristics of children admitted with SARS-CoV-2 pneumonia, identify risk factors associated with severity and compare the cases according to the variant of SARS-CoV-2. This was a descriptive and retrospective study, including patients aged 0–18 years hospitalized in a tertiary-care hospital between 1 March 2020 and 1 March 2022. Epidemiological, clinical, diagnostic and therapeutic data were analyzed. Forty-four patients were admitted; twenty-six (59%) were male and twenty-seven (61%) were older than 12 years. Thirty-six (82%) had comorbidities, the most frequent of which were obesity and asthma. Seven (15.9%) patients required high-flow oxygen, eleven (25%) non-invasive ventilation and four (9.1%) conventional mechanical ventilation. In critically ill patients, higher levels of anemia, lymphopenia, procalcitonin, lactate dehydrogenase (LDH) and hypoalbuminemia and lower levels of HDL-cholesterol were detected (all *p* < 0.05). Prematurity (*p* = 0.022) was associated with intensive care unit admission. Patients were younger during the Omicron wave (*p* < 0.01); no variant was associated with greater severity. In conclusion, pediatric patients with a history of prematurity or with anemia, lymphopenia, elevated procalcitonin, elevated LDH levels, hypoalbuminemia and low HDL-cholesterol levels may require admission and present more severe forms. Apart from age, no notable differences between SARS-CoV-2 variant periods were found.

## 1. Introduction

During the severe acute respiratory syndrome coronavirus 2 (SARS-CoV-2) pandemic, the epidemiology and clinical presentation of COVID-19 in the pediatric population have been largely studied. In general, the literature describes a lower incidence and transmission of the disease in children, together with a lower severity of the clinical course [1,2,3].

Regarding the need for hospitalization in pediatric patients, many studies have focused on describing cases of severe disease in children who require admission to pediatric intensive care units (PICUs), such as multisystem inflammatory syndrome in children (MIS-C) [4]. Given the low global impact of this infection in children and adolescents, there are few studies evaluating the total hospitalization rate in this population, considering both severe and mild cases [5,6].

In addition to MIS-C, other conditions have been described with respiratory involvement in children, such as pneumonia, bronchitis or bronchiolitis [7]. In fact, among hospitalized children with SARS-CoV-2 infection, the most frequent reported diagnosis has been pneumonia [8,9]. However, the clinical characteristics of SARS-CoV-2 pneumonia in pediatric patients are not well defined [10]. In this line, Jiménez-García et al. reported that of all the cases of children admitted with a diagnosis of pneumonia during the first wave of the pandemic in Spain, only 20% were ultimately attributable to SARS-CoV-2 infection [11].

In this sense, it has been difficult to carry out comparative studies because, on the one hand, the overall incidence of SARS-CoV-2 infection in children has been low, and on the other hand, the incidence of community-acquired pneumonia in the pediatric population has decreased drastically, especially at the beginning of the pandemic secondary to isolation measures (lockdowns, use of face masks, social distancing) [12,13]. And finally, with the start of systematic vaccination against SARS-CoV-2, a notable reduction in COVID-19 has also been observed in terms of incidence, hospitalization rate and deaths [14]. The main aim of this study was to describe the characteristics of pediatric patients admitted for SARS-CoV-2 pneumonia, whose diagnostic criteria are not standardized, unlike in the adult population, as well as to identify risk factors associated with greater severity. It must be considered that viral pneumonia in children, especially those under 5 years of age, is a common pathology that often leads to hospitalization. Specifically regarding SARS-CoV-2, children under 5 years of age are not yet eligible to be vaccinated, and therefore are a vulnerable population to SARS-CoV-2. Consequently, it is of interest to be able to provide more information about this pathology in the pediatric population. Furthermore, being able to identify risk factors associated with severity is important to detect early those patients who may have an unfavorable evolution and, therefore, who may benefit from early diagnosis and treatment.

Our secondary objective was to compare the characteristics of the cases according to the predominant variant of SARS-CoV-2 in order to find differences between them.

## 2. Materials and Methods

### 2.1. Design, Subjects and Setting

This was a descriptive and retrospective pilot study. Patients aged 0–18 years admitted to Sant Joan de Déu hospital with SARS-CoV-2 pneumonia between 1 March 2020 and 1 March 2022 were included. This is a university-based, pediatric tertiary care hospital with 345 beds (28 PICU beds), and it is estimated to manage 30% of all annual pediatric hospitalizations in Catalonia (Spain).

All included patients were required to have tested positive for SARS-CoV-2, either by detection of SARS-CoV-2 RNA via real-time reverse transcriptase-polymerase chain reaction (RT-PCR) in nasopharyngeal swab/aspirate (NS/NPA) or saliva, or by detection of SARS-CoV-2 antigen in a nasopharyngeal swab. Respiratory symptoms and radiological pneumonia diagnosis, either by chest X-ray (CXR) or CT-scan, were also required. The exclusion criteria were patients with symptoms or radiological pneumonia diagnosis but isolation of another microorganism or patients with SARS-CoV-2 infection but without associated pneumonia.

In our center, the hospitalization criteria for a patient with SARS-CoV-2 infection were being less than 3 months old (according to their clinical situation), having hypoxemia (SatO_2_ < 92%), having moderate or severe respiratory distress or others (bad general condition, lethargy, refusal to eat, apnea…). In addition to these criteria, admission was assessed for patients with pneumonia with a viral appearance (bilateral consolidation, lymphopenia…) and persistent fever and those patients with severe comorbidities and risk of rapid deterioration.

The criteria for admission to the PICU were severe respiratory distress despite optimizing treatment in the ward, SatO_2_ < 92% with FiO_2_ ≥ 0.5, acute respiratory acidosis (hypercapnia > 55 mmHg and/or pH < 7.3), septic appearance, signs of shock, altered level of consciousness and/or suspicion of hypoventilation of central origin.

The first vaccination campaign against SARS-CoV-2 in Spain for the population between 16 and 29 years old began on 30 June 2021. The campaign for the population between 12 and 15 years old started on 4 August 2021, and the one for the population between 5 and 11 years old started on 15 December 2021 [15].

### 2.2. Variables

Epidemiological, clinical, diagnostic (laboratory and radiographic) and therapeutic data from the electronic medical records were reviewed and incorporated into a database located in a protected folder hosted on the hospital’s servers for subsequent descriptive and comparative analysis. No personal or identifiable data were collected. For the analysis of risk factors, the differences between patients who presented greater severity (PICU admission, greater need for ventilation) and those with a better clinical evolution were analyzed and compared.

RT-PCR of multiple respiratory viruses (apart from SARS-CoV-2) from nasopharyngeal/tracheal aspirate (NPA/TA) or bronchoalveolar lavage (BAL) were conducted for some selected patients in the ward and all those admitted to the PICU to elucidate other etiologies.

Blood tests were performed on all patients at admission and repeated during hospitalization depending on their clinical evolution. Blood cultures were performed in cases of fever, sepsis or worsening of the general condition.

CXR and CT-scans were interpreted by at least two authors to minimize selection bias.

Patients were classified in different periods according to the predominant SARS-CoV-2 variant to analyze if there were differences between them. According to the literature [16,17,18], the periods were defined as: Wuhan period (March 2020–February 2021), Alpha period (B.1.1.7; February 2021–June 2021), Delta period (B.1.617.1; June 2021–December 2021) and Omicron period (B.1.1.529; December 2021–March 2022).

The specific treatment of SARS-CoV-2 pneumonia followed the hospital guidelines and protocols, which were in accordance with the literature. The use of corticosteroids was analyzed separately when indicated as a specific COVID-19 treatment or indicated for associated bronchospasm.

### 2.3. Statistical Analysis and Ethics

Categorical variables were expressed as total number and proportions, while continuous variables were expressed as median and interquartile range (IQR). The comparison of categorical variables was performed using the Chi-Square test and Fisher’s exact test; continuous variables were compared using Student’s t-test and the ANOVA test. The odds ratio, determined with Fisher’s exact test and a 2 × 2 table, was used to assess the association between the risk factors and the severity. All tests were two-sided, and *p*-values less than 0.05 were considered to indicate a statistically significant difference. Statistical analyses and graphing were performed using SPSS^®^, version 25.0.

The study was carried out in accordance with the Helsinki declaration and approved by the Ethical Assistant Committee of Sant Joan de Déu Foundation (code: PIC-90-22; approval date: 12 September 2022). The study followed the requirements of Law 14/2007 of 3 July on Biomedical Research. Informed consent was obtained from all individual participants included in the study or their parents/legal guardians (in the case of children under 16 years old).

## 3. Results

### 3.1. Epidemiological Data

Forty-four patients were admitted with a diagnosis of SARS-CoV-2 pneumonia; twenty-six (59%) were male and the median age was 14 years old (IQR 9–16). Twenty-seven (61%) patients were older than 12 years.

Regarding the distribution of patients in periods according to the predominant SARS-CoV-2 variants, 14 (31.8%) patients were reported in the Wuhan period, 7 (15.9%) in the Alpha period, 11 (25%) in the Delta period and 12 (27.3%) in the Omicron period.

Only two (4.5%) patients were completely vaccinated with two doses of SARS-CoV-2 vaccine at the time of admission.

Thirty-six (82%) patients had at least one comorbidity, and the most frequent were overweight/obesity in thirteen (29.5%) patients and asthma/recurrent wheezing in thirteen (29.5%), followed by other pulmonary and neurological disorders. All the comorbidities as well as other epidemiological data of the patients are reported in Table 1. 

The median days from symptom onset to diagnosis of SARS-CoV-2 infection was 1 day (IQR 0–4), and from symptom onset to SARS-CoV-2 pneumonia diagnosis and hospital admission it was 5.5 days (IQR 3–8). Nasopharyngeal RT-PCR was the most-used diagnostic method in 30 (68%) patients, followed by nasopharyngeal antigen (32%).

Patients had a median hospital stay of 5 days (IQR 3–9). Thirteen (30%) patients were admitted to the PICU, with a median stay at the PICU of 5 days (IQR 4–14) and a median total hospital stay of 13 days (IQR 7–18). 

Regarding complications, four (9.1%) patients had pleural effusion, two (4.5%) had a ventilator-associated pneumonia (VAP) and one (2.3%) had pneumothorax.

No patient presented persistent symptoms or pulmonary sequelae.

Two patients died from COVID-related conditions; both had severe comorbidities. The first patient was a 12-year-old boy with acute lymphocytic leukemia with recurrence who had received allogeneic hematopoietic stem cell transplantation and chimeric antigen receptor T-cell (CAR-T) with secondary complications such as graft-versus-host-disease (GVHD). The second patient was an 8-year-old boy with a history of extreme prematurity, severe neurodevelopmental disorder and recurrent pulmonary infections.

### 3.2. Clinical and Radiologic Data

**Figure 1:**
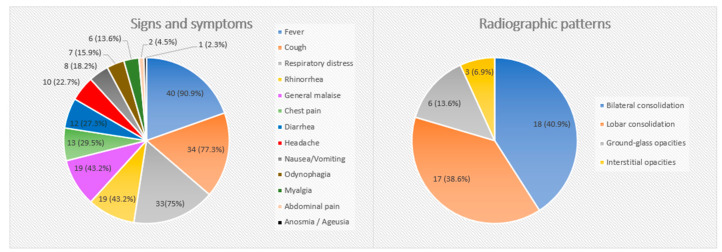
Clinical and radiologic data of the patients. The results are expressed as counts and percentages (in parenthesis).

All patients presented symptoms at admission. The most frequent ones were fever (90.9%), cough (77.3%) and respiratory distress (75%). The median temperature at admission was 39 °C (IQR 38.5–39.5). All the signs and symptoms are described in Figure 1.

Thirty-seven (84%) patients were diagnosed with pneumonia, while seven (16%) had bronchopneumonia.

All patients underwent a chest X-ray (CXR). The most common radiological patterns were bilateral consolidation in 18 (40.9%) patients, followed by lobar consolidation in 17 (38.6%), ground-glass opacities alone in 6 (13.6%) and interstitial opacities in 3 (6.9%). The radiological pattern of bilateral consolidation was more frequently observed in patients older than 12 years of age, without statistically significant differences (14 vs. 4; *p* = 0.409). The distribution of the radiological patterns is described in Figure 1. 

A CT-scan was performed in four patients. In two cases, a CT-Angiography (CTA) was performed to exclude pulmonary thromboembolism due to persistent hypoxemia. The other two cases involved patients receiving immunosuppressive treatment, in whom a CT was requested to evaluate the lung parenchyma in the context of COVID infection, as well as to rule out complications such as a fungal superinfection. All scans showed areas of increased density and ground glass opacities with an alveolar, bilateral and diffuse pattern, predominantly in upper lobes, with no findings of thromboembolism in any case.

### 3.3. Laboratory Data

The results of the blood tests are shown in Table 2. In general, it stands out that the majority of patients had lymphopenia, mild neutrophilia, as well as elevated C-reactive protein (CRP), ferritin, lactate dehydrogenase (LDH), fibrinogen and interleukin-6 (IL-6). They also highlight an elevation of d-dimer and transaminases, as well as hypoalbuminemia and low HDL cholesterol.

In critically ill patients (those admitted to the PICU), higher procalcitonin (PCT) (*p* = 0.019) and LDH levels (*p* = 0.021) were detected, as well as higher anemia (*p* = 0.005), lymphopenia (*p* = 0.002), hypoalbuminemia (*p* = 0.008) and lower HDL cholesterol levels (*p* = 0.017). All the results comparing severity (patients who required admission to the PICU and those who did not) are shown in Table 2.

In patients who required any type of respiratory support (all those who required oxygen), higher lymphopenia (*p* = 0.037) and neutrophilia (*p* = 0.003) were detected, as well as higher C-reactive protein (*p* = 0.046) and aspartate aminotransferase (AST) levels (*p* = 0.009). Patients who required non-invasive ventilation (NIV) had greater anemia (*p* = 0.003) and lymphopenia (*p* = 0.005), higher values of PCT (*p* = 0.008) and LDH (*p* = 0.006) and lower levels of albumin (*p* = 0.008) and HDL cholesterol (*p* = 0.012). Finally, patients who required conventional mechanical ventilation (CMV) had greater anemia (*p* = 0.001) and higher values of ferritin (*p* = 0.002) and triglycerides (*p* = 0.001). All the results comparing the need for respiratory support are shown in the Supplementary Material.

### 3.4. Microbiological Data

Viral coinfections were tested in 20 (45%) patients: RSV was found in three (15%) cases, rhino/enterovirus, influenza B and parainfluenza 3 in one (5%) case and adenovirus in one (5%) case.

Bacterial coinfection was observed in four (9%) patients: one (25%) patient with a history of bronchiectasis had a positive sputum culture for *S. pneumoniae*; one (25%) patient who required NIV had a positive *S. aureus* PCR in the NPA; and two (50%) patients developed VAP due to *S. maltophilia* and *S. marcescens* superinfection, both of which were isolated in a BAL culture.

Lastly, one patient with a single kidney and a history of recurrent urinary tract infections had a positive urine culture for extended-spectrum beta-lactamase (ESBL) *K. pneumoniae* during admission.

Blood cultures were performed in 41 patients, and all of them were negative; PCR for *S. pneumoniae* in blood was performed in 8 patients, and all of them were also negative.

### 3.5. Therapeutical Data

All treatment data are described in Table 3.

Thirty-seven (84.1%) patients required respiratory support. Of the four patients who required CMV, all of them had previously received NIV. The seven patients who did not require respiratory support were high-risk patients with underlying conditions (immunodeficiency, neuromuscular disease…) admitted to the hospital for monitoring and observation or intravenous antibiotic therapy. Two patients required inotropic support for 1–2 days, with a mean Vasoactive Inotropic Score (VIS) of 20 points. No patients required ExtraCorporeal Membrane Oxygenation (ECMO).

### 3.6. Risks Factors Associated with Severity

Among the comorbidities, prematurity was significantly associated with a higher risk of admission to the PICU (OR 13.3; 95%CI 1.3–134.9; *p* = 0.022) and a higher need for advanced respiratory support: NIV (OR 18.2; 95%CI 1.7–189.6; *p* = 0.010) and CMV (OR 57; 95%CI 3.9–825; *p* = 0.003). Neither gender nor any of the age groups were significantly associated with greater severity (need for PICU admission or need for advanced respiratory support). Nor was an association found between viral coinfection, bacterial coinfection, longer time until diagnosis, longer time until admission and greater severity. All these comparisons are described in the Supplementary Material.

### 3.7. Differences Regarding SARS-CoV-2 Variant Periods

Differences between patients according to the periods of the predominant SARS-CoV-2 variants are shown in the Supplementary Material. In the cases reported during the Wuhan, Alpha and Delta periods, the majority of patients were older than 12 years (median age in years: 14.8, 12.7 and 14.4, respectively), while in the Omicron period the majority were younger (median age 6.1 years) (*p* = 0.001). Regarding gender and comorbidities, no statistically significant differences between periods were found.

Regarding symptoms, rhinorrhea was more frequently observed in the Omicron period (*p* = 0.010), diarrhea was more frequently observed in the Wuhan and Alpha periods (*p* = 0.037) and odynophagia was more frequent in the Delta period (*p* = 0.020).

The most frequent radiological patterns according to periods were lobar consolidation and bilateral consolidation in the Wuhan period, interstitial opacities in the Alpha period, bilateral consolidation in the Delta period and lobar consolidation in the Omicron period. These differences were statistically significant (*p* = 0.005).

There were no significant differences in terms of severity factors (greater stay, complications, need for admission to the PICU, respiratory support of any type or treatment with steroids, remdesivir or monoclonal antibodies) among periods.

## 4. Discussion

This article describes the clinical characteristics and risk factors associated with SARS-CoV-2 pneumonia in hospitalized pediatric patients. Several studies have previously reviewed the characteristics of SARS-CoV-2 pneumonia in adults [10], but scarce data are available for the pediatric population. However, pneumonia is one of the main causes of admission in children and adolescents with SARS-CoV-2 infection and is associated with high morbidity [7,9]. This study provides more information about this pathology in the pediatric population, helping to identify patients earlier, especially those who are at greater risk of presenting a torpid evolution, and therefore offering them optimal management, thus improving their prognosis.

SARS-CoV-2 pneumonia in the pediatric population has been more frequently described in adolescents and patients with comorbidities [19,20]. In our study, most patients were older than 12 years and 82% had one or more comorbidities. Our hospital is a tertiary referral center for rare and complex diseases, although it is also a community hospital in the referral area. This may partly explain the high percentage of patients with comorbidity, or it may also indicate that these patients tend to develop more severe pneumonia than the general population. The most frequently observed comorbidities in our study were overweight/obesity and asthma/recurrent wheezing. Obesity has been shown to be a clear risk factor for severe SARS-CoV-2 infection [19,21]. Regarding asthma, it has been associated with a higher rate of hospitalization in children with SARS-CoV-2 infection, but it does not worsen the prognosis of the infection. SARS-CoV-2 also has not been described as a trigger for asthmatic exacerbations [9,22].

Severe COVID-19 is rare in children: PICU admission rates are described around 15% [5,6,9]. In our cohort, the need for PICU admission was double, perhaps overestimated due to the limited sample size and also due to the selection of the sample including only patients with pneumonia (in other series published in our center, the actual PICU admission rate relative to all admitted COVID patients was lower [23,24,25,26]). Fortunately, in the current study the prognosis was found to be favorable, with a low rate of complications and mortality (<5% reported in previous articles; 4.5% in our study); the latter is directly associated with the presence of underlying conditions and not with SARS-CoV-2 as such [27,28].

As in other previously published studies, the most frequent symptoms in our patients were fever, cough and respiratory distress [20,29]. Regarding the radiological characteristics, most of the reviews agree that the findings found in children differ from those found in adults and that it is not necessary to routinely perform a lung CT-scan in addition to a CXR in the pediatric population [30,31,32]. Some authors even propose the extension of the use of lung ultrasound given its great utility without providing radiation [25,33]. In our study, as reported previously [31,32], the most frequent radiological patterns were the presence of consolidation (especially bilateral) and ground glass opacities. 

The role of certain biomarkers in the definition of severe COVID-19 has been investigated, highlighting lymphopenia, acute phase reactants and some cytokines [34,35,36]. In the specific case of COVID-19 pneumonia, even though in the pediatric population the inflammatory response is much lower than in adults [37], the remarkable elevation of cytokines such as IL-6 has been described [38,39]. In our cohort, most patients presented elevated IL-6 values, with a median of 32.1 pg/mL. In relation to this, Shafiek et al. defined the IL-6 cut-off value as a predictor of severe pneumonia in children at 31.7 pg/mL [38]. In fact, in our cohort it was observed that the patients who required admission to the PICU had higher values of IL-6 compared to the rest, although the difference was not statistically significant. On the other hand, in our study patients with severe pneumonia requiring PICU admission had greater anemia and lymphopenia, higher PCT and LDH values, as well as lower levels of albumin and HDL cholesterol. Regarding HDL cholesterol, reviews have recently been published on the role of lipids in SARS-CoV-2 infection in adults, finding that lower levels of total cholesterol, LDL and HDL are associated with greater severity and mortality [40].

As other authors have suggested, ruling out the presence of coinfections with other respiratory viruses should be considered in any child presenting with severe SARS-CoV-2 pneumonia, as it may be useful in its management [41]. In addition, it is considered a challenge to differentiate the signs and symptoms of SARS-CoV-2 pneumonia compared to other viral pneumonias in children, as several researchers have demonstrated [11,28,41]. In our study, even though not all patients were tested, the viral coinfection rate was 11.3%, similar to that reported in the literature. Although probably due to the small sample, no association was found between viral coinfection and greater severity of the patients.

Regarding treatment, like other viral pneumonias, most of the patients in our cohort required respiratory support with low-flow oxygen therapy. Characteristically, 25% required non-invasive ventilation and 9.1% required mechanical ventilation, higher percentages compared to other cases of viral pneumonia, which is why some authors have suggested that the lung damage associated with SARS-CoV-2 is different from that caused by other viruses [28]. On the other hand, 77.3% of the patients required antibiotic treatment for suspected respiratory superinfection. These data may be overestimated, given that all the patients in our sample required hospitalization and therefore were considered more severe than any patient with pneumonia managed in an outpatient setting.

Several risk factors associated with the development of severe disease in pediatric patients with COVID-19 have been described, including age (under 5 years and adolescents); previous comorbidities; symptoms of cough, dyspnea and fever on admission; and lymphopenia, elevated CRP and LDH [34,35,42,43]. Considering some of these variables, Satdhabudha et al. developed a predictive risk score for the development of COVID-19 pneumonia in children. In addition, since this score includes only demographic and clinical variables, the authors suggest that it could reduce the risk of unnecessary radiation exposure in these patients [44]. In our study, the only risk factor associated with greater severity was a history of prematurity, as previously described in the literature [42].

Finally, although it was initially thought that the transmission and impact of SARS-CoV-2 would be lower in the summer season, it was found that the spread of SARS-CoV-2 was independent of the time of year. In our study, the number of patients affected by pneumonia was similar during the four studied periods. Regarding the comparison between periods, we found that the age of the patients in the Omicron period was significantly lower compared to the other periods. These data coincide with other previously published data that showed an increase of up to five times during the Omicron period in the hospitalization rate of children aged 0–4 years old, a population that is not yet eligible for vaccination at this time [45].

Our study has some limitations. In the first place, there are those inherent to a single-center and retrospective study (that limits the ability to establish causality), in addition to the small size of the sample. Similarly, only hospitalized patients were included, so the total incidence of pneumonia due to SARS-CoV-2 may be underestimated, since outpatient pneumonias were not included. In addition, some cases had incomplete data in their medical records, and not all patients underwent a complete blood test or microbiological study. As well, linking a patient to a variant only for the predominant period of the variant, without knowing the true sequence of the virus causing the infection, may be a bias. Finally, the radiographic patterns could have been described differently depending on the observer, raising the possibility of biases.

## 5. Conclusions

In conclusion, pediatric patients with SARS-CoV-2 pneumonia who require admission are usually older than 12 years and have some associated comorbidity. Although most patients require some type of respiratory support, it is generally low-flow oxygen, and the general prognosis is favorable. A history of severe prematurity, anemia, lymphopenia, elevated PCT and LDH and low levels of albumin and HDL cholesterol are associated with an increased risk of severe pneumonia; therefore, the presence of these data should put us on alert in the care of these patients. Regarding the comparison between periods with different predominant SARS-CoV-2 variants, the patients in the Omicron period were younger compared to the other periods. Future studies with a larger number of patients are necessary to validate the results found, in addition to continuing to study the evolution of this disease with future variants of SARS-CoV-2.

## Figures and Tables

**Table 1:** General description of the patients (n = 44).

Epidemiological Data	
Gender (male)	26 (59%)
Age (years)	14 (9–16)
Distribution according to the predominant SARS-CoV-2 variant:	
Wuhan period	14 (31.8%)
Alpha period	7 (15.9%)
Delta period	11 (25%)
Omicron period	12 (27.3%)
Presence of at least one comorbidity	36 (82%)
Comorbidities:	
Obesity/Overweight	13 (29.5%)
Recurrent wheezing/Asthma	13 (29.5%)
Other pulmonary disease	10 (22.7%)
Other neurological disorder	9 (20.5%)
Prematurity ≤ 34 GA	5 (11.4%)
Bronchopulmonary dysplasia	2 (4.5%)
Neuromuscular disorder	4 (9.1%)
Immunodeficiency	4 (9.1%)
Nephropathy	3 (6.8%)
Down syndrome	3 (6.8%)
Cerebral palsy	2 (4.5%)
Cardiopathy	2 (4.5%)
Diabetes mellitus	2 (4.5%)
Other metabolic disorder	2 (4.5%)
Sickle cell anemia	0 (0%)
Other diseases	18 (40.9%)
Hospital stay (days)	5 (3–9)
Admission to the PICU	13 (30%)
PICU stay (days)	5 (4–14)
Complications	7 (15.9%)
Outcome (death)	2 (4.5%)

The results are expressed as counts and percentages (in parenthesis) and as median and interquartile range (25–75) accordingly. GA: gestational age; PICU: pediatric intensive care unit.

**Table 2:** Laboratory results comparing severity (patients who required admission to the PICU and those who did not).

Parameter	Reference Values	All Patients (n = 44)	Patients Admitted to the PICU (n = 13)	Patients Not Admitted to the PICU (n = 31)	*p*-Value
Complete Blood count					
Hemoglobin; minimum (g/dL)	12–16	12.3 (10.8–14.4)	10.6 (9.3–12.6)	13 (11.6–14.7)	0.005
Platelets; minimum (×10^3^/µL)	150–500	181 (140–226)	182 (169–238)	181 (133–225)	0.938
Leucocyte count; maximum (/mm^3^)	5000–12,000	8150 (5850–11,050)	9400 (7400–12,800)	7700 (5600–10,300)	0.320
Lymphocyte count; minimum (/mm^3^)	1400–3300	850 (600–1500)	500 (350–850)	1200 (700–1700)	0.002
Neutrophile count; maximum (/mm^3^)	1500–5000	5750 (3250–7750)	7200 (5000–8500)	4800 (3100–7000)	0.269
Coagulation					
PT; minimum (%)	75–120	79 (72–92)	75 (71–89.5)	81 (72–93)	0.533
aPTT; maximum (seconds)	21–35	26.5 (24.4–29.5)	28.7 (23.5–31.3)	26.5 (24.8–28.9)	0.794
INR; maximum	0.8–1.2	1.12 (1.04–1.20)	1.16 (1.04–1.24)	1.11 (1.04–1.17)	0.305
Fibrinogen; maximum (g/L)	1.5–3.5	4.9 (4.5–6.4)	4.8 (4.2–6.6)	4.9 (4.6–6.4)	0.470
D-dimer; maximum (mg/L)	<0.5	0.9 (0.61–1.94)	1.27 (0.88–2.29)	0.69 (0.42–1.87)	0.613
Biochemistry					
C-reactive protein; maximum (mg/L)	0–15	39.5 (14.7–94)	63.2 (26–156)	35.5 (12–69)	0.216
Procalcitonin; maximum (ng/mL)	<0.5	0.17 (0.06–0.58)	0.52 (0.19–1.5)	0.09 (0.06–0.34)	0.019
Ferritin; maximum (μg/L)	10–120	350.2 (190.7–1246)	1150 (400–1800)	265 (185–685)	0.158
LDH; maximum (IU/L)	<500	837 (621–1093)	930 (800–1900)	790 (580–1050)	0.021
IL-6; maximum (pg/mL)	<5	32.1 (8.5–91.9)	41.9 (26.7–101.9)	9.6 (5.8–129)	0.414
Troponin; maximum (ng/mL)	<0.200	0.002 (0.001–0.007)	0.007 (0.004–0.02)	0.001 (0.0007–0.003)	0.075
NT-ProBNP; maximum (ng/L)	<125	66 (17–163)	196 (93–3400)	19 (14–61)	0.197
Creatin kinase; maximum (UI/L)	64–288	67 (43–144)	57 (28–213)	75 (45–134)	0.467
Sodium; average (mmol/L)	135–145	139 (137–142)	141 (137–145)	139 (136–141)	0.137
Potassium; average (mmol/L)	3.9–5	4.1 (3.8–4.3)	3.9 (3.6–4.1)	4.1 (3.8–4.3)	0.193
Ionic Calcium; average (mmol/L)	1.17–1.30	1.22 (1.15–1.26)	1.23 (1.14–1.28)	1.21 (1.16–1.24)	0.533
Phosphate; average (mg/dL)	3.5–5.7	3.75 (3.15–4.3)	3.75 (2.87–4.65)	3.8 (3.2–4.3)	0.954
Magnesium; average (mg/dL)	1.8–2.3	2.1 (1.8–2.2)	2 (1.7–2.3)	2.1 (1.8–2.2)	0.406
Creatinine; maximum (mg/dL)	<0.90	0.59 (0.47–0.72)	0.58 (0.46–0.81)	0.59 (0.46–0.72)	0.153
AST; maximum (IU/L)	2–38	48 (30–93)	54 (33–123)	46 (30–63)	0.800
ALT; maximum (IU/L)	2–31	39 (22–105)	59 (22–106)	36 (21–116)	0.511
Total Bilirubin; maximum (mg/dL)	0.2–1	0.35 (0.3–0.57)	0.5 (0.3–0.85)	0.35 (0.27–0.52)	0.234
Conjugated Bilirubin, maximum (mg/dL)	<0.2	0.2 (0.1–0.3)	0.2 (0.1–0.3)	0.2 (0.1–0.25)	0.329
Albumin; minimum (mg/dL)	37–54	33 (28–37)	29 (28–32)	37 (35–38)	0.008
Triglycerides; average (mg/dL)	38–161	125 (73–286)	205 (75–327)	105 (57–170)	0.159
Total cholesterol; average (mg/dL)	95–201	129 (89–157)	129 (94–161)	121 (85–160)	0.888
HDL cholesterol; average (mg/dL)	>40	26 (21–32)	23 (17–26)	30 (24–38)	0.017
LDL cholesterol; average (mg/dL)	<147	85 (62–97)	87 (62–101)	81 (50–97)	0.922
VLDL cholesterol; average (mg/dL)	0–24	22 (14–41)	28 (15–54)	18 (12–28)	0.359

The results are expressed as median and interquartile range (25–75). PICU: pediatric intensive care unit, CRP: C-reactive protein, PCT: procalcitonin, LDH: lactate dehydrogenase, IL-6: interleukin-6, PT: prothrombin time, aPTT: activated partial thromboplastin time, INR: international normalized ratio, NT-ProBNP: N-terminal pro brain natriuretic peptide, CK: creatin kinase, AST: aspartate aminotransferase, ALT: alanine aminotransferase, HDL cholesterol: high-density lipoprotein cholesterol, LDL cholesterol: low-density lipoprotein cholesterol, VLDL cholesterol: very low-density lipoprotein cholesterol.

**Table 3:** Treatment of the patients.

Treatment	Patients; n (%)	Duration; Days (IQR)
Respiratory support		
Low-flow oxygen (LFO)	34 (77.3%)	3 (2–5)
High-flow oxygen (HFO)	7 (15.9%)	2 (1–4)
Non-invasive ventilation (NIV)	11 (25%)	4 (3–5)
Conventional mechanical ventilation (CMV)	4 (9.1%)	8.5 (7–12)
Corticosteroids (used as COVID-19 treatment)		
Methylprednisolone	14 (48.3%)	
Dexamethasone	13 (44.8%)	
Hydrocortisone	1 (3.4%)	
Prednisone	1 (3.4%)	
Total ^1^	29/44 (65.9%)	5 (3–10)
Antibiotics		
Respiratory superinfection suspicion	34 (77.3%)	7 (3–10)
Other superinfection suspicion (e.g., UTI)	1 (2.3%)	10
Azithromycin (COVID targeted treatment) ^2^	6 (13.6%)	5
Antivirals		
Remdesivir	7 (15.9%)	5 (5–7)
Immunomodulators		
Tocilizumab	4 (9.1%)	1
Other monoclonal antibodies (Siltuximab, Anakinra)	1 (2.3%)	1
Other treatments		
Heparin (prophylaxis)	33 (75%)	
Hydroxychloroquine ^3^	6 (13.6%)	

^1^ Intravenous: 28 (96.6%), oral: 1 (3.4%). ^2,3^ It was only used in the Wuhan period. IQR: interquartile range; UTI: urinary tract infection.

## Data Availability

The datasets generated during and/or analyzed during the current study are not publicly available due to the fact that individual privacy could be compromised, but they are available from the corresponding author on reasonable request.

## Supplementary Materials

The following supporting information can be downloaded at https://www.mdpi.com/article/10.3390/children10101703/s1, Table S1: Laboratory results comparing patients who required any type of respiratory support with those who did not and patients who required NIV and CMV with those who did not; Table S2: Evaluation of potential risk factors associated with greater severity: admission to the PICU, need for NIV and need for CMV; Table S3: Differences of the patients regarding SARS-CoV-2 variant periods.

## References

[1] Castagnoli R., Votto M., Licari A., Brambilla I., Bruno R., Perlini S., Rovida F., Baldanti F., Marseglia G.L. (2020). Severe Acute Respiratory Syndrome Coronavirus 2 (SARS-CoV-2) Infection in Children and Adolescents. JAMA Pediatr..

[2] Gaythorpe K.A.M., Bhatia S., Mangal T., Unwin H.J.T., Imai N., Cuomo-Dannenburg G., Walters C.E., Jauneikaite E., Bayley H., Kont M.D. (2021). Children’s role in the COVID-19 pandemic: A systematic review of early surveillance data on susceptibility, severity, and transmissibility. Sci. Rep..

[3] Chakkour M., Salami A., Olleik D., Kamal I., Noureddine F.Y., El Roz A., Ghssein G. (2022). Risk Markers of COVID-19, a Study from South-Lebanon. COVID.

[4] García-Salido A., Vicente J.C.D.C., Hofheinz S.B., Ramírez J.B., Barrio M.S., Gordillo I.L., Yuste A.H., Pardellans C.G., Tejedor M.C.-M., Labarga B.H. (2020). Severe manifestations of SARS-CoV-2 in children and adolescents: From COVID-19 pneumonia to multisystem inflammatory syndrome: A multicentre study in pediatric intensive care units in Spain. Crit. Care.

[5] Kim T.Y., Kim E.C., Agudelo A.Z., Friedman L. (2021). COVID-19 hospitalization rate in children across a private hospital network in the United States: COVID-19 hospitalization rate in children. Arch. Pediatr..

[6] Uka A., Buettcher M., Bernhard-Stirnemann S., Fougère Y., Moussaoui D., Kottanattu L., Wagner N., Zimmermann P., Ritz N., Albisetti M. (2022). Factors associated with hospital and intensive care admission in paediatric SARS-CoV-2 infection: A prospective nationwide observational cohort study. Eur. J. Pediatr..

[7] Andina-Martinez D., Alonso-Cadenas J.A., Cobos-Carrascosa E., Bodegas I., Oltra-Benavent M., Plazaola A., Epalza C., Jimenez-García R., Moraleda C., Tagarro A. (2022). SARS-CoV-2 acute bronchiolitis in hospitalized children: Neither frequent nor more severe. Pediatr. Pulmonol..

[8] Parisi G.F., Indolfi C., Decimo F., Leonardi S., del Giudice M.M. (2020). COVID-19 Pneumonia in Children: From Etiology to Management. Front. Pediatr..

[9] Tagarro A., Cobos-Carrascosa E., Villaverde S., Sanz-Santaeufemia F.-J., Grasa C., Soriano-Arandes A., Hernanz A., Navarro M.L., Pino R., Epalza C. (2022). Clinical spectrum of COVID-19 and risk factors associated with severity in Spanish children. Eur. J. Pediatr..

[10] Li J., He X., Yuan Y., Zhang W., Li X., Zhang Y., Li S., Guan C., Gao Z., Dong G. (2021). Meta-analysis investigating the relationship between clinical features, outcomes, and severity of severe acute respiratory syndrome coronavirus 2 (SARS-CoV-2) pneumonia. Am. J. Infect. Control..

[11] Jimenez-García R., Nogueira J., Retuerta-Oliva A., Sainz T., Cano-Fernández J., Flores-Pérez P., Méndez-Echevarría A., Villalobos-Pinto E., Calleja-Gero L., Sanz-Santaeufemia F.J. (2021). Pneumonia in Hospitalized Children during SARS-CoV-2 Pandemic. Is It All COVID-19? Comparison between COVID and Non-COVID Pneumonia. Pediatr. Infect. Dis. J..

[12] Rybak A., Yang D.D., Schrimpf C., Guedj R., Levy C., Cohen R., Gajdos V., Tort J., Skurnik D., Ouldali N. (2021). Fall of Community-Acquired Pneumonia in Children following COVID-19 Non-Pharmaceutical Interventions: A Time Series Analysis. Pathogens.

[13] Noureddine F.Y., Chakkour M., El Roz A., Reda J., Al Sahily R., Assi A., Joma M., Salami H., Hashem S.J., Harb B. (2021). The emergence of SARS-CoV-2 variant(s) and its impact on the prevalence of COVID-19 cases in the Nabatieh Region, Lebanon. Med. Sci..

[14] Borchering R.K., Mullany L.C., Howerton E., Chinazzi M., Smith C.P., Qin M., Reich N.G., Contamin L., Levander J., Kerr J. (2023). Impact of SARS-CoV-2 vaccination of children ages 5–11 years on COVID-19 disease burden and resilience to new variants in the United States, November 2021–March 2022: A multi-model study. Lancet Reg. Health Am..

[15] Gobierno de España Estrategia de Vacunación COVID-19. https://www.vacunacovid.gob.es/.

[16] Secretaria de Salut Pública—Departament de Salut—Generalitat de Catalunya (2021). Informe de Vigilancia de las Variantes Genómicas del Coronavirus SARS-CoV-2 en Cataluña. Semana 49—2021 (6 Diciembre 2021–12 Diciembre 2021). Casos Notificados al Sistema de Notificación Microbiológica de Cataluña (SNMC). https://scientiasalut.gencat.cat/bitstream/handle/11351/6958/informe_vigilancia_variants_genomiques_coronavirus_sars_cov2_setmana_49_2021_cas.pdf?sequence=2&isAllowed=y.

[17] Secretaria de Salut Pública—Departament de Salut—Generalitat de Catalunya (2022). Informe de Vigilancia de Las Variantes Genómicas Del Coronavirus SARS-CoV-2 En Cataluña. Semana 9—2022 (28 Febrero–6 Marzo 2022). Casos Notificados al Sistema de Notificación Microbiológica de Cataluña (SNMC). https://scientiasalut.gencat.cat/bitstream/handle/11351/8609/informe_vigilancia_variants_genomiques_coronavirus_sars_cov2_setmana_9_2022_cas.pdf?sequence=2&isAllowed=y.

[18] Secretaria de Salut Pública—Departament de Salut—Generalitat de Catalunya (2021). Informe de Vigilancia de Las Variantes Genómicas Del Coronavirus SARS-CoV-2 En Cataluña, Semana 22—2021 (31/05/2021 al 06/06/2021). Casos Notificados al Sistema de Notificación Microbiológica de Cataluña (SNMC). https://scientiasalut.gencat.cat/bitstream/handle/11351/6583/informe_vigilancia_variants_genomiques_coronavirus_sars_cov2_setmana_22_2021_cas.pdf?sequence=2&isAllowed=y.

[19] Moreno-Noguez M., Rivas-Ruiz R., Roy-García I.A., Pacheco-Rosas D.O., Moreno-Espinoza S., Flores-Pulido A.A. (2021). Risk factors associated with SARS-CoV-2 pneumonia in the pediatric population. Bol. Med. Hosp. Infant. Mex..

[20] Ozcan S., Emeksiz S., Perk O., Uyar E., Yüksek S.K. (2021). Severe Coronavirus Disease Pneumonia in Pediatric Patients in a Referral Hospital. J. Trop. Pediatr..

[21] Chao J.Y., Sugarman A., Kimura A., Flamer S., Jing T.T., Fernandes D.M., Khine H., Shinnar S., Lo Y., Cabana M.D. (2022). Factors Associated with Hospitalization in Children and Adolescents with SARS-CoV-2 Infection. Clin. Pediatr..

[22] Gaietto K., Freeman M.C., DiCicco L.A., Rauenswinter S., Squire J.R., Aldewereld Z., Iagnemma J., Campfield B.T., Wolfson D., Kazmerski T.M. (2022). Asthma as a risk factor for hospitalization in children with COVID-19: A nested case-control study. Pediatr. Allergy Immunol..

[23] Melé M., Henares D., Pino R., Asenjo S., Matamoros R., Fumadó V., Fortuny C., García-García J.-J., Jordan I., Brotons P. (2021). Low impact of SARS-CoV-2 infection among paediatric acute respiratory disease hospitalizations. J. Infect..

[24] Girona-Alarcon M., Bobillo-Perez S., Sole-Ribalta A., Hernandez L., Guitart C., Suarez R., Balaguer M., Cambra F.-J., Jordan I., KIDS-Corona study group (2021). The different manifestations of COVID-19 in adults and children: A cohort study in an intensive care unit. BMC Infect. Dis..

[25] Guitart C., Suárez R., Girona M., Bobillo-Perez S., Hernández L., Balaguer M., Cambra F.J., Jordan I. (2021). Lung ultrasound findings in pediatric patients with COVID-19. Eur. J. Pediatr..

[26] Ríos-Barnés M., Lanaspa M., Noguera-Julian A., Baleta L., De Sevilla M.F., Ferri D., Götzens J., Jordan I., Lecina L., Monfort L. (2021). The Spectrum of COVID-19 Disease in Adolescents. Arch. Bronconeumol..

[27] Wei J.S. (2020). How lethal is SARS-CoV-2 pneumonia when compared with respiratory syncytial virus and influenza in young children?. Aust. J. Gen. Pract..

[28] del Valle R., Ballesteros Á., Calvo C., Sainz T., Mendez A., Grasa C., Molino P.R., Mellado M.J., Sanz-Santaeufemia F.J., Herrero B. (2022). Comparison of pneumonia features in children caused by SARS-CoV-2 and other viral respiratory pathogens. Pediatr. Pulmonol..

[29] Yurtseven A., Turan C., Özenen G.G., Işik H., Bal Z., Sertöz R., Saz E.U. (2022). Characteristics of pediatric COVID-19 patients admitted to the emergency department and factors associated with pneumonia. Turk. J. Emerg. Med..

[30] Palabiyik F., Kokurcan S.O., Hatipoglu N., Cebeci S.O., Inci E. (2020). Imaging of COVID-19 pneumonia in children. Br. J. Radiol..

[31] Liu H., Liu F., Li J., Zhang T., Wang D., Lan W. (2020). Clinical and CT imaging features of the COVID-19 pneumonia: Focus on pregnant women and children. J. Infect..

[32] Kurian J., Blumfield E., Levin T.L., Liszewski M.C. (2022). Imaging findings in acute pediatric coronavirus disease 2019 (COVID-19) pneumonia and multisystem inflammatory syndrome in children (MIS-C). Pediatr. Radiol..

[33] Roychowdhoury S., Bhakta S., Mahapatra M.K., Ghosh S., Saha S., Konar M.C., Sarkar M., Nandi M. (2022). Role of lung ultrasound patterns in monitoring coronavirus disease 2019 pneumonia and acute respiratory distress syndrome in children. Clin. Exp. Pediatr..

[34] Tsabouri S., Makis A., Kosmeri C., Siomou E. (2021). Risk Factors for Severity in Children with Coronavirus Disease 2019: A Comprehensive Literature Review. Pediatr. Clin. N. Am..

[35] Zhou B., Yuan Y., Wang S., Zhang Z., Yang M., Deng X., Niu W. (2021). Risk profiles of severe illness in children with COVID-19: A meta-analysis of individual patients. Pediatr. Res..

[36] Li Y., Deng W., Xiong H., Li H., Chen Z., Nie Y., Wang Z., Li K., Li J. (2020). Immune-related factors associated with pneumonia in 127 children with coronavirus disease 2019 in Wuhan. Pediatr. Pulmonol..

[37] Eshkiki Z.S., Shahriari A., Seyedtabib M., Torabizadeh M., Assarehzadegan M.A., Nashibi R., Khosravi M., Neisi N., Mard S.A., Shayesteh A.A. (2021). Innate and Adaptive Immunity Imbalance with Severe COVID-19 Pneumonia in Children and Adults. Front. Pediatr..

[38] Shafiek H.K., El Lateef H.M.A., Boraey N.F., Nashat M., Abd-Elrehim G.A.B., Abouzeid H., Hafez S.F.M., Shehata H., Elhewala A.A., Abdel-Aziz A. (2021). Cytokine profile in Egyptian children and adolescents with COVID-19 pneumonia: A multicenter study. Pediatr. Pulmonol..

[39] Maggio M.C., Failla M.C., Giordano S., La Manna M.P., Sireci G. (2022). Interleukin-6 Is a Promising Marker of COVID-19 in Children: A Case Series of 2 Brothers with Severe COVID-19 Pneumonia. Am. J. Case Rep..

[40] Chidambaram V., Geetha H.S., Kumar A., Majella M.G., Sivakumar R.K., Voruganti D., Mehta J.L., Karakousis P.C. (2022). Association of Lipid Levels with COVID-19 Infection, Disease Severity and Mortality: A Systematic Review and Meta-Analysis. Front. Cardiovasc. Med..

[41] Ng D.C.-E., Tan K.K., Ting G.S.S., Ling C., Fadzilah N.F.B., Tan S.F., Subramaniam T., Zailanalhuddin N.E.B., Lim H.Y., Baharuddin S.B. (2022). Comparison of Severe Viral Pneumonia Caused by SARS-CoV-2 and Other Respiratory Viruses among Malaysian Children During the COVID-19 Pandemic. Front. Pediatr..

[42] Graff K., Smith C., Silveira L., Jung S., Curran-Hays S., Jarjour J., Carpenter L.B., Pickard K.B., Mattiucci M., Fresia J.B. (2021). Risk Factors for Severe COVID-19 in Children. Pediatr. Infect. Dis. J..

[43] Mania A., Faltin K., Mazur-Melewska K., Małecki P., Jończyk-Potoczna K., Lubarski K., Lewandowska Z., Cwalińska A., Rosada-Kurasińska J., Bartkowska-Śniatkowska A. (2021). Clinical picture and risk factors of severe respiratory symptoms in COVID-19 in children. Viruses.

[44] Satdhabudha A., Chaiyakulsil C., Uppala R., Niyomkarn W., Tovichien P., Norasettekul V., Ruangnapa K., Smathakanee C., Choursamran B., Kulbun A. (2022). Development and validation of the predictive score for pediatric COVID-19 pneumonia: A nationwide, multicenter study. PLoS ONE.

[45] Marks K.J., Whitaker M., Agathis N.T., Anglin O., Milucky J., Patel K., Pham H., Kirley P.D., Kawasaki B., Meek J. (2022). Hospitalization of Infants and Children Aged 0–4 Years with Laboratory-Confirmed COVID-19—COVID-NET, 14 States, March 2020–February 2022. MMWR. Morb. Mortal. Wkly. Rep..

