# Supplementary material for: Characteristics and Risk Factors Associated with SARS-CoV-2 Pneumonias in Hospitalized Pediatric Patients: A Pilot Study
Source: Children (Basel). 2023 Oct 19;10(10):1703. doi: 10.3390/children10101703 (PMC10605629; doi:10.3390/children10101703)
Supplement: Supplementary file 1 [file children-10-01703-s001.zip › children-2616095-supplementary.pdf]

Table S1: Laboratory results comparing patients who required any type of respiratory support with those who did not, and patients who required NIV and CMV with those who did not. The results are expressed as median and interquartile range [25-75].

Parameter	Patients who required any type respiratory support (n=37)	Patients who DID NOT required respiratory support (n=7)	p-value	Patients who required NIV (n=11)	Patients DID NOT required NIV (n=33)	p-value	Patients who required CMV (n=4)	Patients who DID NOT required CMV (n=40)	p-value
Hemoglobin; minimum (g/dL)	12.4 [10.8 – 14.6]	**11.8 [10.8 – 13]**	0.572	**10.6 [9.4 – 12.2]**	13 [11.5 – 14.6]	**0.003**	**9.3 [9 – 10.3]**	12.7 [11.4 – 14.5]	**0.001**
Platelets; minimum (x10³/µl)	181 [163 – 226]	147 [121 – 321]	0.961	182 [168 – 227]	181 [135 – 234]	0.808	182 [92 – 258]	179 [140 – 226]	0.750
Leucocyte count; maximum (/mm3)	9200 [6500 – 11400]	5600 [3800 – 8200]	**0.021**	10900 [7400 – 13000]	7700 [5500 – 10200]	0.118	11000 [4400 – 17000]	8050 [5800 – 10800]	0.547
Lymphocyte count; minimum (/mm3)	**800 [550 – 1400]**	1600 [600 – 2000]	**0.037**	**600 [400 – 900]**	**1100 [700 – 1600]**	**0.005**	**700 [150 – 1400]**	**850 [620 – 1500]**	0.188
Neutrophile count; maximum (/mm3)	**6100 [3900 – 8000]**	3000 [2200 – 5800]	**0.003**	**7800 [5200 – 8600]**	4800 [3050 – 6900]	0.110	**6500 [2500 – 10100]**	**5700 [3200 – 7500]**	0.841
C-reactive protein; maximum (mg/L)	**49 [16 – 112]**	**17 [2.8 – 69]**	**0.046**	**63 [31 – 178]**	**35 [13 – 71]**	0.192	**46 [16 – 148]**	**39 [14 – 94]**	0.988
Procalcitonin; maximum (ng/mL)	0.18 [0.06 – 0.57]	0.12 [0.05 – 0.62]	0.124	**0.52 [0.31 – 1.64]**	0.09 [0.06 – 0.38]	**0.008**	**0.72 [0.36 – 1.26]**	0.11 [0.06- 0.54]	0.731
Ferritin; maximum (ug/L)	**400 [190 – 1300]**	**263 [136 – 310]**	0.258	**819 [385 – 1952]**	**268 [189 – 742]**	0.112	**1990 [166 – 9400]**	**310 [190 – 1150]**	**0.002**
LDH; maximum (IU/L)	**840 [720 – 1090]**	**613 [589 – 949]**	0.074	**1086 [805 – 2123]**	**791 [589 – 1029]**	**0.006**	**1960 [1050 – 3100]**	**832 [612 – 1070]**	0.132
IL-6; maximum (pg/mL)	**32 [8.5 – 91]**	**20 [3 – 40]**	0.191	**43 [25 – 112]**	**11 [6 – 85]**	0.379	**71 [4.6 – 123]**	**28 [9 – 94]**	0.458
PT; minimum (%)	76 [71 – 91]	85 [76 – 98]	0.351	75 [69 – 82]	81 [72 – 93]	0.130	79 [74 – 94]	79 [71 – 92]	0.700
aPTT; maximum (seconds)	26 [23 – 29]	28 [25 – 29]	0.494	25 [22 – 31]	26 [24 – 28]	0.830	28 [18 – 32]	26 [24 – 29]	0.945
INR; maximum	1.13 [1.04 – 1.21]	1.11 [1.01 – 1.19]	0.443	1.18 [1.08 – 1.29]	1.11 [1.03 – 1.15]	0.113	1.10 [0.9 – 1.20]	1.13 [1.05 – 1.23]	0.381
Fibrinogen; maximum (g/L)	**5.3 [4.6 – 6.5]**	**4.5 [3.9 – 6]**	0.605	**5.1 [4.4 – 6.8]**	**4.9 [4.5 - 6.1]**	0.969	**4.3 [3.7 – 5.5]**	**5 [4.6 – 6.7]**	0.069
D-dimer;	**1 [0.6 – 2.1]**	0.42 [0.39 – 1.92]	0.162	**1.2 [0.8 – 2.4]**	**0.75 [0.42 – 1.8]**	0.687	**2.1 [1.1 – 3.4]**	**0.89 [0.58 – 1.86]**	0.750

maximum (mg/L)									
Troponin; maximum (ng/mL)	0.002 [0.001 – 0.007]	0.002 [0.0002 – 0.011]	0.234	0.007 [0.005 – 0.085]	0.001 [0.001 – 0.003]	**0.041**	0.007 [0.001 – 0.02]	0.002 [0.001 – 0.006]	**0.003**
NT-ProBNP; maximum (ng/L)	81 [16 – 196]	20 [14 – 90]	0.731	**160 [131 – 262]**	20 [15 – 81]	0.101	**162 [135 – 196]**	36 [15 – 87]	0.494
Creatin kinase; maximum (UI/L)	67 [45 – 161]	71 [13 – 180]	0.345	53 [25 -215]	75 [46 – 130]	0.238	140 [24 – 450]	67 [45 – 134]	**0.001**
Sodium; average (mmol/L)	140 [136 -143]	138 [137 – 141]	0.164	141 [139 – 146]	138 [136 – 141]	0.052	141 [139 – 148]	139 [136 – 141]	0.289
Potassium; average (mmol/L)	4 [3.8 – 4.2]	4.3 [3.6 – 4.5]	0.652	4 [3.6 – 4.2]	4.1 [3.8 – 4.3]	0.332	3.8 [3.5 – 4.6]	4.1 [3.8 – 4.3]	0.844
Ionic Calcium; average (mmol/L)	1.22 [1.15 – 1.26]	1.20 [1.17 – 1.26]	0.984	1.23 [1.14 – 1.29]	1.21 [1.18 – 1.25]	0.533	1.29 [1.14 – 1.32]	1.21 [1.15 – 1.25]	0.127
Phosphate; average (mg/dL)	3.7 [3.2 – 4.3]	3.7 [3.1 – 4]	0.915	3.7 [2.5 – 4.9]	3.7 [3.1 – 4.2]	0.776	5 [3.8 – 5.2]	3.7 [3.1 – 4.3]	0.456
Magnesium; average (mg/dL)	2.1 [1.8 – 2.2]	1.9 [1.8 – 2.1]	0.093	2.1 [1.8 – 2.6]	2 [1.8 – 2.1]	0.128	2.2 [2 – 2.3]	2.05 [1.8 – 2.1]	0.370
Creatinine; maximum (mg/dL)	0.62 [0.51 – 0.74]	0.46 [0.43 – 0.60]	**0.009**	0.58 [0.46 – 0.72]	0.59 [0.47 – 0.72]	0.148	0.59 [0.42 – 1.85]	0.59 [0.47 – 0.72]	0.053
AST; maximum (IU/L)	**53 [30 – 102]**	**39 [28 – 48]**	**0.009**	**91 [33 – 138]**	**44 [30 – 63]**	0.509	**72 [54 – 126]**	**45 [30 – 74]**	0.698
ALT; maximum (IU/L)	**46 [21 – 112]**	26 [21 – 77]	0.170	**81 [24 – 119]**	31 [20 – 104]	0.777	**76 [35 – 112]**	**34 [20 – 105]**	0.637
Total Bilirubin; maximum (mg/dL)	0.4 [0.3 – 0.6]	0.3 [0.2 – 0.4]	0.113	0.9 [0.3 – 1.2]	0.3 [0.3 – 0.5]	0.238	0.6 [0.3 – 1.3]	0.3 [0.3 – 0.5]	0.180
Conjugated Bilirubin, maximum (mg/dL)	0.2 [0.1 – 0.3]	0.1 [0.1 – 0.2]	0.186	0.2 [0.1 – 0.3]	0.2 [0.1 – 0.2]	0.332	0.2 [0.1 – 0.3]	0.2 [0.1 – 0.3]	0.504
Albumin; minimum (mg/dL)	**30 [28 – 36]**	**36 [35 – 40]**	0.113	**29 [28 – 32]**	37 [35 – 42]	**0.008**	**30 [28 – 32]**	**36 [29 – 38]**	0.180
Triglycerides; average (mg/dL)	125 [75 – 288]	124 [70 – 190]	0.399	**176 [75 – 345]**	115 [61 – 206]	0.149	**240 [201 – 381]**	113 [71 – 220]	**0.001**
Total cholesterol; average (mg/dL)	131 [91 – 164]	95 [77 – 140]	0.223	132 [92 – 166]	123 [86 – 156]	0.854	150 [123 – 183]	123 [87 – 149]	0.299
HDL cholesterol; average (mg/dL)	**26 [23 – 31]**	**27 [16 – 34]**	0.938	**23 [17 – 25]**	**29 [25 – 38]**	**0.012**	**22 [17 – 29]**	**26 [23 – 33]**	0.566

LDL cholesterol; average (mg/dL)	86 [64 – 101]	56 [44 – 120]	0.252	89 [65 – 105]	77 [52 – 95]	0.718	105 [90 – 132]	81 [56 – 91]	0.115
VLDL cholesterol; average (mg/dL)	23 [15 – 47]	15 [14 – 20]	**0.009**	**25 [15 – 55]**	19 [12 – 38]	0.585	**30 [28 – 52]**	19 [14 – 35]	0.478

NIV : non-invasive ventilation, CMV : conventional mechanical ventilation, CRP : C-reactive protein, PCT : procalcitonin, LDH : lactate dehydrogenase, IL-6 : interleukin-6, PT : prothrombin time, aPTT : activated partial thromboplastin time, INR : international normalized ratio, NT-ProBNP: N-terminal pro brain natriuretic peptide, CK : creatin kinase, AST : aspartate aminotransferase, ALT : alanine aminotransferase, HDL cholesterol : high-density lipoprotein cholesterol, LDL cholesterol : low-density lipoprotein cholesterol, VLDL cholesterol : very low-density lipoprotein cholesterol.

Table S2: Evaluation of potential risk factors associated with greater severity: admission to the PICU, need for NIV and need for CMV. The results are expressed as counts and percentages (in parenthesis).

**Risk factor**	**Patients ADMITTED to the PICU (n=13)**	**Patients NOT admitted to the PICU (n=31)**	**Odds Ratio [95% CI]**	**p-value**	**Patients who required NIV (n=11)**	**Patients who DID NOT required NIV (n=33)**	**Odds Ratio [95% CI]**	**p-value**	**Patients who required CMV (n=4)**	**Patients who DID NOT required CMV (n=40)**	**Odds Ratio [95% CI]**	**p-value**
Gender (males)	9 (69.2%)	17 (54.8%)	0.54 [0.13 – 2.13]	0.376	7 (63.6%)	19 (57.6%)	0.77 [0.18 – 3.17]	1	3 (75%)	23 (57.5%)	0.45 [0.04 – 4.72]	0.634
Gender (females)	4 (30.8%)	14 (45.2%)			4 (36.4%)	14 (42.4%)			1 (25%)	17 (42.5%)		
Age (<12 years old)	6 (46.2%)	10 (32.3%)	0.55 [0.14 – 2.09]	0.496	5 (45.5%)	11 (33.3%)	0.6 [0.14 – 2.4]	0.492	3 (75%)	13 (32.5%)	0.16 [0.01 – 1.69]	0.129
Age (≥12 years old)	7 (53.8%)	21 (67.7%)			6 (54.5%)	22 (66.7%)			1 (25%)	27 (67.5%)		
Presence of comorbidities	11 (84.6%)	25 (80.6%)	1.32 [0.22 – 7.59]	1	9 (81.8%)	27 (81.8%)	1 [0.17 – 5.86]	1	4 (100%)	32 (80%)		1
Obesity / Overweight	3 (23.1%)	10 (32.3%)	0.63 [0.14 – 2.8]	0.722	2 (18.2%)	11 (33.3%)	0.44 [0.08 – 2.4]	0.461	0	13 (32.5%)		
Recurrent wheezing / Asthma	1 (7.7%)	12 (38.7%)	0.13 [0.01 – 1.14]	0.068	1 (9.1%)	12 (36.4%)	0.17 [0.02 – 1.54]	0.132	0	13 (32.5%)		
Other pulmonary disease	4 (30.8%)	6 (19.4%)	1.85 [0.42 – 8.11]	0.449	4 (36.4%)	6 (18.2%)	2.57 [0.56 – 11.6]	0.237	2 (50%)	8 (20%)	4 [0.48 – 32.9]	0.218
Other neurological disorder	2 (15.4%)	7 (22.6%)	0.62 [0.11 – 3.5]	0.703	1 (9.1%)	8 (24.2%)	0.31 [0.03 – 2.83]	0.411	0	9 (22.5%)		
Prematurity ≤ 34 GA	4 (30.8%)	1 (3.2%)	**13.3 [1.3 – 134.9]**	**0.022**	4 (36.4%)	1 (3%)	**18.2 [1.7 – 189.6]**	**0.010**	3 (75%)	2 (5%)	**57 [3.9 – 825]**	**0.003**
Neuromuscular disorder	3 (23.1%)	1 (3.2%)	9 [0.83 – 96.6]	0.071	2 (18.2%)	2 (6.1%)	3.4 [0.42 – 28]	0.256	0	4 (10%)		
Immunodeficiency	2 (15.4%)	2 (6.5%)	2.6 [0.33 – 21.1]	0.570	1 (9.1%)	3 (9.1%)	1 [0.93 – 10.7]	1	1 (25%)	3 (7.5%)	4.1 [0.32 – 52.6]	0.327
Nephropathy	1 (7.7%)	2 (6.5%)	1.2 [0.1 – 14.6]	1	0	3 (9.1%)			0	3 (7.5%)		
Down syndrome	1 (7.7%)	2 (6.5%)	1.2 [0.1 – 14.6]	1	1 (9.1%)	2 (6.1%)	1.55 [0.1 – 18.9]	1	1 (25%)	2 (5%)	6.33 [0.43 – 91.7]	0.254
Cerebral palsy	1 (7.7%)	1 (3.2%)	2.5 [0.14 – 43.2]	0.508	1 (9.1%)	1 (3%)	3.2 [0.18 – 55.9]	0.442	0	2 (5%)		
Cardiopathy	1 (7.7%)	1 (3.2%)	2.5 [0.14 – 43.2]	0.508	1 (9.1%)	1 (3%)	3.2 [0.18 – 55.9]	0.442	0	2 (5%)		

Diabetes mellitus	2 (15.4%)	0			1 (9.1%)	1 (3%)	3.2 [0.18 – 55.9]	0.442	1 (25%)	1 (2.5%)	13 [0.64 – 263.8]	0.175
Other metabolic disorder	1 (7.7%)	1 (3.2%)	2.5 [0.14 – 43.2]	0.508	1 (9.1%)	1 (3%)	3.2 [0.18 – 55.9]	0.442	1 (25%)	1 (2.5%)	13 [0.64 – 263.8]	0.175
Other diseases	6 (46.2%)	12 (38.7%)	1.3 [0.36 – 5]	0.647	4 (36.4%)	14 (42.4%)	0.77 [0.18 – 3.17]	1	1 (25%)	17 (42.5%)	0.45 [0.43 – 4.7]	0.634
Time until diagnosis ≤2 days (n=30)	7 (23.3%)	23 (76.6%)	2.46 [0.63 – 9.55]	0.288	5 (16.7%)	25 (83.3%)	3.7 [0.89 – 15.6]	0.132	2 (6.7%)	28 (93.3%)	2.33 [0.29 – 18.5]	0.581
Time until diagnosis >2 days (n=14)	6 (42.9%)	8 (57.1%)			6 (42.9%)	8 (57.1%)			2 (14.3%)	12 (85.7%)		
Time until admission ≤6 days (n=25)	10 (40%)	15 (60%)	0.28 [0.06 – 1.22]	0.081	8 (32%)	17 (68%)	0.39 [0.09 – 1.77]	0.301	4 (16%)	21 (84%)		0.122
Time until admission >6 days (n=19)	3 (15.8%)	16 (84.2%)			3 (15.8%)	16 (84.2%)			0	0		
Viral coinfection	3 (23.1%)	2 (6.4%)	3 [0.37 – 24.17]	0.347	3 (27.2%)	2 (6.1%)	3 [0.37 – 24.17]	0.347	2 (50%)	3 (18.8%)	4.3 [0.4 – 44.4]	0.249
Bacterial coinfection	3 (23.1%)	1 (3.2%)		1	3 (27.2%)	1 (3%)	1 [0.4 – 24.5]	1	2 (50%)	2 (5%)	1 [0.63 – 15.9]	1

PICU : pediatric intensive care unit, NIV : non-invasive ventilation, CMV : conventional mechanical ventilation, GA : gestational age.

Table S3: Differences of the patients regarding SARS-CoV-2 variant periods: Wuhan period (March 2020-February 2021), Alpha period (B.1.1.7; February 2021-June 2021), Delta period (B.1.617.1; June 2021 - December 2021) and Omicron period (B.1.1.529; December 2021 - March 2022). The results are expressed as counts and percentages (in parenthesis).

**Variable**	**Wuhan period (n=14)**	**Alpha period (n=7)**	**Delta period (n=11)**	**Omicron period (n=12)**	**p-value**
Gender (males)	8 (57.1%)	5 (71.4%)	7 (63.6%)	6 (50%)	0.809
Gender (females)	6 (42.9%)	2 (28.6%)	4 (36.4%)	6 (50%)	
Age (<12 years old)	3 (21.4%)	2 (28.6%)	1 (9.1%)	10 (83.3%)	**0.001**
Age (≥12 years old)	11 (78.6%)	5 (71.4%)	10 (90.9%)	2 (16.7%)	
Presence of comorbidities	10 (71.4%)	7 (100%)	9 (81.8%)	10 (83.3%)	0.459
Obesity / Overweight	6 (42.9%)	2 (28.6%)	4 (36.4%)	1 (8.3%)	0.258
Recurrent wheezing / Asthma	3 (21.4%)	1 (14.3%)	4 (36.4%)	5 (41.7%)	0.509
Other pulmonary disease	3 (21.4%)	1 (14.3%)	2 (18.2%)	4 (33.3%)	0.754
Other neurological disorder	3 (21.4%)	3 (42.9%)	2 (18.2%)	1 (8.3%)	0.350
Prematurity ≤ 34 GA	1 (7.1%)	0	2 (18.2%)	2 (16.7%)	0.575
Neuromuscular disorder	2 (14.3%)	1 (14.3%)	1 (9.1%)	0	0.596
Immunodeficiency	3 (21.4%)	1 (14.3%)	0	0	0.164
Nephropathy	1 (7.1%)	1 (14.3%)	0	1 (8.3%)	0.690
Down syndrome	0	0	2 (18.2%)	1 (8.3%)	0.282
Cerebral palsy	0	0	1 (9.1%)	1 (8.3%)	0.589
Cardiopathy	0	0	0	2 (16.7%)	0.134
Diabetes mellitus	2 (14.3%)	0	0	0	0.213
Other metabolic disorder	0	1 (14.3%)	1 (9.1%)	0	0.349
Other diseases	6 (42.9%)	6 (85.7%)	1 (9.1%)	5 (41.7%)	**0.015**
Fever	14 (100%)	6 (85.7%)	9 (81.8%)	11 (91.7%)	0.434
Cough	9 (64.3%)	4 (57.1%)	9 (81.8%)	12 (100%)	0.085
Respiratory distress	10 (71.4%)	5 (71.4%)	10 (90.9%)	8 (66.7%)	0.558
Rhinorrhea	3 (21.4%)	2 (28.6%)	4 (36.4%)	10 (83.3%)	**0.010**
General malaise	5 (35.7%)	3 (42.9%)	7 (63.6%)	4 (33.3%)	0.446
Chest pain	4 (28.6%)	3 (42.9%)	4 (36.4%)	2 (16.7%)	0.614
Diarrhea	7 (50%)	3 (42.9%)	1 (9.1%)	1 (8.3%)	**0.037**
Headache	5 (35.7%)	2 (28.6%)	3 (27.3%)	0	0.162
Nausea/Vomiting	2 (14.3%)	1 (14.3%)	0	5 (41.7%)	0.069
Odynophagia	1 (7.1%)	0	5 (45.5%)	1 (8.3%)	**0.020**

Myalgia	3 (21.4%)	1 (14.3%)	2 (18.2%)	0	0.422
Abdominal pain	1 (7.1%)	1 (14.3%)	0	0	0.416
Anosmia / Ageusia	1 (7.1%)	0	0	0	0.533
CXR: Bilateral consolidation	6 (42.9%)	1 (14.3%)	8 (72.7%)	3 (25%)	**0.005**
CXR: Lobar consolidation	6 (42.9%)	2 (28.6%)	2 (18.2%)	7 (58.3%)	
CXR: Ground-glass opacities	2 (14.3%)	1 (14.3%)	1 (9.1%)	2 (16.7%)	
CXR: Interstitial opacities	0	3 (42.9%)	0	0	
Total hospital stay ≤5 days (n=22)	5 (22.7%)	4 (18.2%)	7 (31.8%)	6 (27.3%)	0.551
Total hospital stay >5 days (n=22)	9 (40.9%)	3 (13.6%)	4 (36.4%)	6 (27.3%)	
Admission to the PICU	4 (28.6%)	2 (28.6%)	4 (36.4%)	3 (25%)	0.945
Total PICU stay ≤5 days (n=9)	3 (33.3%)	2 (22.2%)	3 (44.4%)	1 (11.1%)	0.419
Total PICU stay >5 days (n=4)	1 (25%)	0	1 (25%)	2 (50%)	
Presenting complications	2 (14.3%)	1 (14.3%)	1 (9.1%)	4 (33.3%)	0.761
Need for respiratory support	11 (78.6%)	6 (85.7%)	10 (90.9%)	10 (83.3%)	0.868
Need for NIV	3 (21.4%)	1 (14.3%)	4 (36.4%)	3 (25%)	0.734
Need for CMV	1 (7.1%)	0	1 (9.1%)	2 (16.7%)	0.660
Treatment with steroids	8 (57.1%)	6 (85.7%)	9 (81.8%)	6 (50%)	0.232
Treatment with remdesivir	1 (7.1%)	1 (14.3%)	2 (18.2%)	3 (25%)	0.659
Treatment with immunomodulators	3 (21.4%)	0	1 (9.1%)	0	0.534

GA : gestational age, CXR : chest X-ray, PICU : pediatric intensive care unit, NIV : non-invasive ventilation, CMV : conventional mechanical ventilation.
